# The History of Medicine and Surgery in Georgia

**Published:** 1895-04

**Authors:** Luther B. Grandy

**Affiliations:** Atlanta, Ga.


ATLANTA

Medical and Surgical Journal.

VOL. XII. APRIL, 1895. No. 2.

LUTHER B. GRANDY, M.D., MANAGING EDITOR. MILLER B. HUTCHINS, M.D., BUSINESS MANAGER.

### ORIGINAL COMMUNICATIONS.

### THE HISTORY OF MEDICINE AND SURGERY IN GEORGIA.*

BY LUTHER B. GRANDY, M.D.,
ATLANTA, GA.

Dr. Louis A. Dugas, of Augusta, was a great surgeon, fertile in invention, bold and successful in execution. Before beginning the practice of medicine he had enjoyed three years of the best surgical advantages under such European masters as Velpeau, Dupuytren, Larrey, Lisfranc, Civiale, and others. Though possessed of a versatile genius and a wide range of medical knowledge, his professional life was devoted especially to surgery. He was one of the founders of the Medical College of Georgia, and occupied the chair of surgery for twenty-five years (1855-1880).

While in Paris in 1828, Dr. Dugas saw Civiale demonstrate his new method of performing lithotrity. He witnessed the development of the operation abroad, and upon his return home (1831) he became one of its earliest advocates and representatives in America.

Allusion has been made above to his "operations during mes-

*Continued from page 716, February number.

meric insensibility." I attempted also to give some idea of the attention which this subject was receiving at that time from scientific men (1835-1845). Dr. Dugas was the first in this country to apply the test of a surgical operation to mesmerism. His first operation was done January 12, 1845, Professors Ford, Means, and Garvin assisting. The patient, Mrs. Clarke, was mesmerized, and "two elliptical incisions about eight inches in length were made, comprehending between them the nipple and a considerable portion of skin; the integuments were dissected up in the usual manner and the entire mamma removed. It weighed sixteen ounces. . . . . The ordinary dressing was applied. . . . . During the operation the patient gave no indication whatever of sensibility. . . . . She remained in the same sound and quiet sleep as before the use of the knife. . . . . When she awoke and was told that the breast had been removed she expressed great incredulity," etc. There was a rapid recurrence of this tumor, and the second growth was removed in the same manner, June 13. Another recurrence was noticed in a few months, this time involving the axillary glands. The third operation was done November 11, with the extirpation of the enlarged glands. In both these operations the patient was unconscious and insensitive as before.

Dr. Dugas believed that mesmerism was a reality, and he hoped that the phenomena of mesmeric sleep would be so studied and utilized as to "render them available to all sufferers."* The introduction of anesthesia by ether doubtless stopped further experiments of this character.

Dislocations of the shoulder-joint were once difficult of diagnosis, from lack of definite indications. Dr. Dugas, in 1856, first called attention to a diagnostic symptom, simple but certain.† It has since been known as "Dugas' sign." This sign was: "If the fingers of the injured limb can be placed upon the sound shoulder, while the elbow touches the thorax, there can be no dislocation; and if this cannot be done there must be a dislocation. It is physically impossible to bring the elbow in contact with the ster-

*_Southern Medical and Surgical Journal_, March, May, September, 1845, February, 1846.

†_Southern Medical and Surgical Journal_, March, 1856. Also Transactions American Medica Association, 1857.

num or front of the thorax if there be a dislocation; and the inability to do this is proof positive of dislocation."

No subject is of more interest to the present-day surgeon than the treatment of penetrating wounds of the abdomen. Up to ten or twelve years ago the usual treatment of these wounds, Gross says, was "hasty, careless, and slovenly." It is ordinarily taught that Dr. Marion Sims was the first to advise opening the abdomen at once to locate and suture wounds in the intestines. But this procedure, which was advised by Sims in connection with the special case of President Garfield, in the summer of 1881, had been recommended as a general surgical principle five years before by Dr. Dugas.* At the International Medical Congress in Philadelphia, 1876, in the surgical section, Dr. Dugas read a paper on this subject which was a clear and forcible argument for a more enlightened, rational, and radical treatment of these cases. His paper was entitled, "Remarks upon Penetrating Wounds of the Abdomen, with the Suggestion of a Change of Practice in such Cases." He said: "The awful fatality of penetrating wounds of the abdomen would seem to indicate the necessity for a change in practice, . . . . and I now make a formal appeal to the profession for a reconsideration of the principles by which we have been governed in such cases, and to suggest that in all instances of penetrating wounds of the abdomen in which there may be any suspicion of the effusion of blood or feces into the peritoneal cavity, the surgeon should proceed immediately as follows: (1) Induce anesthesia; (2) lay open the abdomen along the linea alba freely enough to make a thorough inspection of the parts contained; (3) ligate all bleeding vessels as far as possible; (4) examine carefully the whole length of the alimentary canal, in order to detect any wounds, and to stitch them; (5) in gunshot wounds reduce the channels of entrance and exit through the abdominal walls to the form of incised wounds; (6) cleanse the wound and peritoneum of all extraneous materials, and close the abdominal walls with suitable sutures," etc. He and others had "successfully carried out these principles in cases in which the wounded intestines had protruded." "Why,"

*One case is reported by Kinloch, of Charleston, in which this principle was successfully practiced during the late war. See *American Journal of Medical Sciences*, July, 1867.

he asked, "should we allow other cases to die simply because the abdominal wound is not large enough to permit the bowels to come into view?"

In spite of a busy practice, and professorial and editorial duties, Dr. Dugas contributed more than one hundred medical papers to current literature. Besides those above alluded to, there were: "Report of Surgical Cases," "Anatomy and Pathology of the Liver," "Glanders in the Human Subject," "The Treatment of Fractures," "Lithotrity for Urinary Calculus," "Ligation of the Common Iliac Artery for Aneurism of the Sciatic," "The Pathology and Treatment of Convulsions," "Army Diseases," "Pathological Peculiarities of the Negro Race," "Cases of Lithotomy," "Relative Value of Lithotrity and Lithotomy," "Wounds of the Small Intestines," "Use of Quinine in Intermittent and Remittent Fever," etc.

After 1850 there came a period of especial activity in medical teaching. One College had held the field for over twenty years.

The Savannah Medical College had been incorporated as far back as 1838, but a faculty was not organized until 1853. The first course of lectures was delivered in 1853-54 by the following faculty: R. D. Arnold, M.D., Professor of the Theory and Practice of Medicine; W. G. Bullock, M.D., Professor of the Principles and Practice of Surgery; J. G. Howard, M.D., Professor of Anatomy; P. M. Kollock, M.D., Professor of Obstetrics and Diseases of Women and Children; H. L. Byrd, M.D., Professor of Materia Medica and Therapeutics; E. H. Martin, M.D., Proefssor of Physiology; C. W. West, M.D., Professor of Chemistry; J. B. Read, M.D., Professor of Pathological Anatomy. The exercises were continued regularly every year from 1853 to 1861, inclusive, when the outbreak of the civil war brought about a suspension. The faculty was reorganized in 1867, and the college exercises continued regularly until March, 1881, when, in consequence of the loss by death of several members of the faculty and the lack of sufficient encouragement, the college again suspended and has so remained up to the present time.

In 1855 the Oglethorpe Medical College was organized in Savannah and the first session opened in November. The faculty consisted of: Dr. Jas. S. Morel, Professor of Anatomy; Dr. H. L. Byrd, Professor of the Practice of Medicine; Dr. LeRoy Antony, Professor of Obstetrics and Diseases of Women; Dr. W. C. Norwood, Professor of Materia Medica and Therapeutics; Dr. Wm. T. Feay, Professor of Chemistry and Pharmacy; Dr. Chas. Ganahl, Professor of Surgery. With some changes in this faculty the college taught until the spring of 1861. After the war it was not reopened.

In the summer of 1854 the first steps were taken to establish a college in Atlanta. After some months consumed in the careful selection of a faculty the Atlanta Medical College opened in May, 1855, with the following corps of teachers: Dr. M. G. Slaughter, Professor of Anatomy; Dr. J. W. Jones, Professor of the Practice of Medicine; Dr. Jesse Boring, Professor of Obstetrics and Diseases of Women and Children; Dr. W. F. Westmoreland, Professor of Surgery; Dr. J. E. Dubose, Professor of Physiology; Dr. G. T. Wilburn, Professor of Surgical and Pathological Anatomy; Dr. J. J. Robertson, Professor of Chemistry and Medical Jurisprudence; Dr. J. G. Westmoreland, Professor of Materia Medica and Therapeutics, and Dean of Faculty. Some changes were soon made in the faculty. Professor Slaughter was succeeded by Dr. H. W. Brown, Professor Boring by Dr. Thomas S. Powell, Professor Dubose by Dr. Jos. P. Logan, and Professor Robertson by Dr. Alexander Means, who had been Professor of Chemistry in the College at Augusta. With this faculty the college continued in successful operation up to the outbreak of war (1861). For four years the college taught only during the summer session (May to August inclusive). In November, 1858, a new and important feature was added: a winter course, preparatory to the regular summer course, lasting from November to the end of February. The close of the war found the College property almost in ruins. The work of rehabilitation was undertaken with energy and success by the two Doctors Westmoreland, and lectures were resumed in the autumn of 1865.

The Reform College (Macon) continued in operation (except during the war) until 1874, when its name was changed to the College of American Medicine and Surgery. Six professors constituted the faculty; two lived in Macon, one in Atlanta, two in New York, and one in Michigan. In 1881 the college was moved to Atlanta. In 1877 the Georgia Eclectic Medical College was launched in Atlanta, and in 1884 this institution and the preceding united their fortunes under the name of the Georgia College of Eclectic Medicine and Surgery.

THE ATLANTA MEDICAL AND SURGICAL JOURNAL was inaugurated as an adjunct of the Atlanta Medical College at the close of the first session. The first number appeared September, 1855, under the management of Professors W. F. Westmoreland and J. P. Logan. It was published regularly until the summer of 1861, when it was suspended by war. After the war, it pursued an uncertain career, witnessing two or three periods of suspended animation, with as many resuscitations, and many changes of editors. In March, 1884, it again resumed publication, and has appeared without intermission since that time. For many years this journal was the organ of the College, edited by members of the faculty and supported and cherished largely by the alumni.

Dr. J. B. Bean, of Macon, divides with Dr. Gunning, of New York, the credit for inventing and introducing the interdental splint of vulcanized rubber for the treatment of fractures of the lower jaw. This vulcanite splint, made from plaster casts of the teeth and gums, was a decided improvement upon the appliances formerly used for these fractures. Dr. Bean used his interdental splint during the late war in about forty cases of fracture of the lower jaw. Results were highly satisfactory. (*Richmond Medical Journal*, February, 1866.) The apparatuses of Bean and Gunning were devised about the same time and entirely independently of each other.

Dr. Robert Battey, of Rome, was the first surgeon in the State to do an ovariotomy. This was in 1867. A thirty pound dermoid cyst was completely and successfully removed, the patient being the wife of a physician in Alabama. She is still living.

Dr. Battey's famous operation on August 17, 1872, was the first ovariotomy done in Georgia. It was devised and practiced "with a view to establish at once the change of life for the effectual remedy of certain otherwise incurable maladies." (*Atlanta Medical and Surgical Journal*, September, 1872.) It was advocated by the author only as a last resort, after the failure of all other means of relieving certain disorders. The operation was generally condemned in vigorous terms, both at home and abroad. But professional sentiment gradually changed, the first recognition and encouragement coming from the Boston Gynecological Society. Next, Dr. Thomas, of New York, indorsed it in his work on diseases of women. Then Sims, who in 1872 had been "astounded at the audacity of this operation," said in 1877 that Dr. Battey had "reasoned out a truism in seeking to effect a change of life by extirpating the ovaries; that his operation was based upon sound physiological doctrine, and that it accomplished in practice just what it proposed to do." When the operation thus became a well-recognized surgical procedure, claims for priority were entered by Hegar of Germany and Tait of England; but they were not thoroughly substantiated, and to Dr. Battey properly belongs the credit of introducing the operation and of the moral courage involved in it. The operation has been much abused. The best description of its true indications is Dr. Battey's paper before the American Gynecological Seciety, 1880, entitled "The Proper Field for Battey's Operation."

The Southern Medical College was inaugurated in 1879, and the first session opened in October of that year. Some months were consumed in the selection of a permanent faculty which resulted in the following appointments: Thomas S. Powell, M.D., Professor of Obstetrics and Diseases of Women; William Perrin Nicolson, M.D., Professor of Anatomy; R. C. Word, M.D., Professor of Physiology; John Thad Johnson, M.D., Professor of Surgery; G. G. Roy, M.D., Professor of Materia Medica and Therapeutics; Albert S. Payne, M.D., Professor of the Practice of Medicine; and E. J. Hallock, M.D., Professor of Chemistry. In 1885 Dr. J. McFadden Gaston became Professor of Surgery; Dr. Word was succeeded by Dr. John C. Olmsted and Dr. F. W. McRae, and Dr. Payne was succeeded by Dr. W. D. Bizzell and Dr. Jas. B. Baird. The Southern College has taught without interruption since its organization.

The revival of the supra-pubic operation for stone in the bladder is recent medical history, dating from 1880, and due principally to two German surgeons. The method had been practiced many times by the older American surgeons, but it gradually fell into disuse, not to say disrepute; and it is said* that in the year 1875 there were "comparatively few medical men who had ever seen it done," and "little was known of it by men of considerable eminence in the profession." One of the first to practice it about this time was Dr. E. F. Starr, of Nacoochee. In May, 1876, he did the operation with success, removing a stone which weighed nine drachms. The bladder was not injected with fluid; no drainage was used; the wounds in the bladder and abdominal wall were closed with the same set of sutures. (See *American Journal of Medical Sciences*, July, 1877.)

The name and reputation of Dr. Willis F. Westmoreland are too well known, particularly to the Georgia profession, to require much space here. Dr. Westmoreland, soon after his graduation, placed himself under the special instruction of Dr. Paul F. Eve, and later he became for three years a pupil of Velpeau, Nelaton, Ricord, and others in Paris. He was thus a surgeon by training, and made more so by study and experience. While abroad, in 1855, he was elected to the chair of surgery in the Atlanta Medical College, a position which he honorably filled for the remainder of his life. In 1855 he and Dr. Joseph P. Logan organized the *Atlanta Medical and Surgical Journal.* It was an adjunct of the college, and ably conducted. During the war both college and *Journal* went down, and Dr. Westmoreland gave his services to the Confederacy. In 1865 he was active in the reorganization of both, and was identified with both to the day of his death. Dr. Westmoreland combined happily the two most essential qualities of a good surgeon—operative skill and accurate powers of diagnosis. He wrote little, so the work of a lifetime, rich in valuable surgical experience and full of honors and successes, unfortunately is not of record. Most of his papers are to be found in the transactions of the Medical Association and in the *Atlanta Medical and Surgical Journal.* Dr. Westmoreland died in June, 1889, from the effect of a wound received in operating.

*Dr. C. W. Dulles, *American Journal Medical Sciences*, July, 1875.

Surgery of the gall-bladder has been very much enriched by the experimental studies of Dr. J. McFadden Gaston of Atlanta.. Up to 1880 Sims and Tait had successfully treated cholelithiasis and obstructions of the bile ducts by opening the gall-bladder through the abdominal wall. The results of this cholecystotomy were not wholly satsfactory, from the establishment of biliary fistulæ, more or less permanent, and the necessary loss of bile to the system. In 1884 Dr. Gaston began a series of experiments on twenty-one dogs, with the view of establishing a communication between the gall-bladder and duodenum, in order that the bile might be preserved for physiological purposes. These experiments were highly encouraging, and convinced Dr. Gaston of the feasibility of the operation to which he gave the name *duodeno-cholecystostomy*. Results of these experiments were published in the *Atlanta Medical and Surgical Journal* (September, October, 1884; also, September, November, 1885). A paper was also published by Dr. Gaston in Gaillard's *Medical Journal*, October, 1884, entitled, "Obstruction of the Gall-duct and its Consequences, with Remedial Operation Suggested." The same issue of the last named journal said editorially that the proposed method was "great, original, and ingenious, creating a new, bold, and life-saving operation." Acting upon these experiments and suggestions, a number of surgeons in this country and abroad have operated upon the human subject, and now duodeno-cholecystostomy (or cholecysto-enterostomy) occupies a well-recognized place in abdominal surgery.*

A few words in closing, to complete the history of medical legislation. As above stated, the act of 1825, repealed in 1836, was revived in 1839. It was further re-enacted, without alteration or modification, in 1847. Between this date and 1880 the history of the "board of physicians" was one of decline and inaction. The objects and aims for which the board was constituted simply went by default, because it seemed to be nobody's business to see that the law was carried out. The operations of the board, such as they were, were marked by the most flagrant abuses. Licenses to practice were issued to parties possessing absolutely no intellectual or professional qualifications, the only requirement seem-

* The article on "Gall-Bladder Surgery," by Dr. Gaston, in the supplement to the Reference Hand-book of Medical Sciences (1893), will be interesting in this connection.

ing to be the license fee of five dollars. Thus the law was more honored in the breach than in the observance, and it thus stood for years upon our statute books as only a mockery of decent medical legislation. The result was that quacks and imcompetents of every breed and variety found no difficulty in making a home in Georgia. The Medical Association of the State in 1881 took steps to correct these evils, and presented a bill to the legislature for this purpose. This bill, designed to be radical and thorough, was so mutilated by amendments in the legislature as to be absolutely worthless. Friends of the measure then accepted as next best the "Medical Practice Act of 1881," which simply required that applicants, to practice medicine in the State, should be graduates of an incorporated medical college, school, or university, and that they should record their diplomas with the county clerks, etc. The gain thus accomplished was more apparent than real, since county clerks could have no knowledge of the value of different diplomas, whether bogus or genuine, or tell the difference between a medical diploma and a Latin encyclical from the vatican. So also, the purposes of this law were defeated, and quacks who would not be admitted into other States continued to find easy entrance into Georgia. In November, 1892, steps were again taken to remove the evils, this time originating in the Atlanta Society of Medicine. A bill for a board of medical examiners was presented to the legislature by a committee of the society, but nothing was accomplished at this session. By the next meeting of the legislature, the movement had received the indorsement and co-operation of the State Association and prominent individual physicians. The bill presented by these gentlemen provided for a board of examiners, the different schools—regular, eclectic, and homeopathic—to be represented upon it in approximate proportion to their numerical strength in the State. The bill was vigorously fought before the judiciary committee of the house by the eclectics, homeopaths, and their hired attorneys. These introduced a substitute bill, providing for three separate boards—regular, eclectic, and homeopathic. Even this was defeated when put upon its passage. In the summer of 1894 another bill was prepared, based upon the above substitute bill. It was intended to be a measure upon which all could agree, regardless of school. It received the indorsement of representative eclectics and homeopaths in Atlanta, and they gave it their active support before the legislature November, 1894. With two or three trivial amendments, it became a law practically without opposition, and went into effect January 1, 1895. Three boards of examiners, consisting of five members each, were appointed by the governor, and thus for the first time in our medical history we had a medical law which promised to be effective, because it recognized individual fitness as determined by personal examination as the only proper qualification for practice.

---

In the preceding pages I have attempted to bring together only the most important items of our medical history. Reference has been had, in the main, to original work and observations by our physicians and surgeons. To discuss all the individuals who have been prominent and successful in their professional relations would have taken one far beyond the limits of this paper. It is hoped that whatever this article lacks in completeness will be supplied by some one else, in order that a tangible and enduring record may be made of the contributions of our medical men to our medical history.

In closing I must acknowledge my indebtedness to Drs. Eugene Foster and Jos. Eve Allen, of Augusta, J. C. LeHardy and Wm. Duncan, of Savannah, Robert Battey, of Rome, H. V. M. Miller, W. P. Nicolson, and W. F. Westmoreland, of Atlanta, and others, for information and assistance.

